# Diagnostic Performance of AST Scale in Mexican Male Population for Alcohol Withdrawal Syndrome

**DOI:** 10.3390/ijerph19159208

**Published:** 2022-07-28

**Authors:** Juan Antonio Suárez-Cuenca, Christian Gabriel Toledo-Lozano, Maryjose Daniela Espinosa-Arroyo, Nallely Alejandra Vázquez-Aguirre, Gandhy Thomas Fonseca-González, Karen Garro-Almendaro, Alberto Melchor-López, Victor Hugo García-López, Abril Ortiz-Matamoros, Tania Ortega-Rosas, Sofia Lizeth Alcaraz-Estrada, Paul Mondragón-Terán, Silvia García

**Affiliations:** 1 Internal Medicine Department, Hospital General Xoco, SEDESA, Mexico City 03330, Mexico; suarej05@gmail.com (J.A.S.-C.); mespinosa1112@gmail.com (M.D.E.-A.); crisrubis@hotmail.com (N.A.V.-A.); karga.fate@gmail.com (K.G.-A.); dralbertomelchor@gmail.com (A.M.-L.); 2 Department of Clinical Research, Centro Médico Nacional “20 de Noviembre”, ISSSTE, Mexico City 03229, Mexico; abrilom@gmail.com (A.O.-M.); ainattt@hotmail.com (T.O.-R.); 3 Nephrology Department, Centro Médico Nacional “20 de Noviembre”, ISSSTE, Mexico City 03229, Mexico; thom_fons@hotmail.com; 4 Internal Medicine Department, Hospital General Tláhuac, SEDESA, Mexico City 13278, Mexico; meladoc_1999@yahoo.com; 5 Department of Genomic Medicine, Centro Médico Nacional “20 de Noviembre”, ISSSTE, Mexico City 03229, Mexico; sofializeth@gmail.com; 6 Coordination of Research, Centro Médico Nacional “20 de Noviembre”, ISSSTE, Mexico City 03229, Mexico; p.mondragonteran@gmail.com

**Keywords:** alcohol withdrawal syndrome, AST scale, diagnostic performance, GMAWS, CIWA-Ar

## Abstract

Alcohol withdrawal syndrome (AWS) represents an adverse consequence of chronic alcohol use that may lead to serious complications. Therefore, AWS requires timely attention based on its early recognition, where easy-to-apply diagnostic tools are desirable. Our aim was to characterize the performance of a short-scale AST (Anxiety, Sweats, Tremors) in patients from public general hospitals. We conducted a cross-sectional study of patients attended at the Emergency Department diagnosed with AWS. Three scales were applied: CIWA-Ar (Clinical Institute Retirement Assessment Scale-Revised), GMAWS (Glasgow Modified Alcohol Withdrawal Syndrome) and AST. Cronbach’s alpha and Cohen’s kappa tests were used for reliability and concordance. Factorial analysis and diagnostic performance including ROC curve were carried out. Sixty-eight males with a mean age of 41.2 years old, with high school education and robust alcohol consumption, were included. Mean scores for CIWA-Ar, GMWAS and AST were 17.4 ± 11.2, 3.9 ± 2.3 and 3.8 ± 2.6, respectively, without significant differences. The AST scale showed an acceptable reliability and concordance (0.852 and 0.439; *p* < 0.0001) compared with CIWA-Ar and GMAWS. AST component analysis evidenced tremor (77.5% variance), sweat (12.1% variance) and anxiety (10.4% variance). Diagnostic performance of the AST scale was similar to the GMAWS scale, evidencing a sensitivity of 84%, specificity of 83.3% and Area Under the Curve (AUC) of 0.837 to discriminate severe AWS, according to CIWA-Ar. The performance of the AST scale to evaluate AWS is comparable with the commonly used CIWA-Ar and GMAWS scales. AST further represents an easy-to-apply instrument.

## 1. Introduction

Alcohol is one of the most consumed psychoactive substances. It causes dependence, and its excessive consumption is linked to several organic and mental diseases [1,2,3]. Alcohol use disorders (AUD) affect 76.3 million people worldwide and are responsible for nearly 3 million deaths, attributed to the harmful use of alcohol [4,5]. Based on the results of a US national survey conducted in 2014, more than half of the population had consumed alcohol in the previous 30 days, of which 44% had met the criteria of excessive alcohol consumption [6,7]. In Mexico, alcohol consumption is characterized as excessive [8]. According to the 2016 National Survey on Drug, Alcohol and Tobacco Consumption (ENCODAT), 71% of the population has consumed alcohol at least once in their life and 33.6% reported excessive consumption in the last year, while alcohol consumption has increased significantly in subjects younger than 18 years old [9].

Globally, alcohol use is one of the main causes of years of life lost due to premature death, morbidity and mortality [1,10]. Excessive alcohol consumption has been causally linked to more than 60 different medical conditions that result in an increased risk of hospitalization [11,12]. It is estimated that between 10% and 33% of patients admitted to Intensive Care Units (ICU) have AUD [13], and one out of four patients admitted to medical and surgical services in general hospitals suffers from an AUD [14]. On the other hand, AUDs are commonly underdiagnosed, maybe because patients tend to underestimate or deny alcohol use when asked by doctors. This fact represents a risk of developing withdrawal symptoms during hospitalization accompanied by the potential worsening of their clinical condition and prognosis [15,16].

Alcohol withdrawal syndrome (AWS) is one of the adverse consequences of chronic alcohol use. It has a wide spectrum of symptoms, ranging from insignificant discomfort to a life-threatening syndrome with delirium, seizures and serious neurological complications [17,18,19]. AWS can evolve within a few hours or days after a sudden cessation or reduction in alcohol intake and is characterized by hyperactivity of the autonomic nervous system that results in the development of typical symptoms [20]. Severe cases of AWS require ICU admission [21], and its severity is associated with length of stay and mortality [20,21,22,23]. It has been estimated that approximately 50% of heavy alcohol users experience withdrawal symptoms when they reduce or stop their consumption [20,21,22,23] and approximately 10% experience withdrawal seizures [23,24,25]. When AWS progresses to a state of severe confusion and hallucinations associated with severe autonomic hyperactivity, what has been called delirium tremens occurs [23,24,26], which carries a higher rate of mortality from 1% to 5%. Therefore, AWS represents a medical emergency [22] and early recognition is relevant for hospitalized patients, since symptoms may be misinterpreted as part of current medical or surgical conditions, leading to inappropriate therapy [14].

Taking into account the prevalence of AWS, the potential severity of its manifestations and the fact that it is a common problem in the field of hospitalized patients, efforts have been made to develop tools that allow the early identification of people at higher risk and their optimal treatment [27,28,29]. Thus far, the standard of care involves providing a symptom-triggered dose of benzodiazepine treatment [22,30,31,32] and the use of an alcohol withdrawal severity scale to define treatment. Among the available scales, the current “gold standard” is the Clinical Institute Withdrawal Assessment Alcohol Scale (CIWA-A) [33,34], which after its creation was modified to reduce the number of items (from 15 to 10), giving rise to the CIWA-A revised (CIWA-Ar), which has documented validity and is the most widely used version [34,35,36,37].

Although the use of the CIWA-Ar is prevalent, other tools have recently been developed to assess the severity of AWS, which vary in terms of the type and number of symptoms included, as well as their versatility for application [38]. One of the scales is the Glasgow Modified Alcohol Withdrawal Syndrome (GMAWS) score, which consists of five items: tremor, sweating, hallucination, orientation, and agitation [39]. Several studies have reported an acceptable agreement between GMAWS and CIWA-Ar, with the latter being preferred due to its ease of use [29]. However, both scales include a large number of items, representing a longer time for their application, and options for each item may result ambiguous, giving rise to a subjective assessment leading to the variability of results between evaluators [40,41,42].

There has been a continuous interest to develop shorter, easier and more reliable scales to evaluate AWS [43,44,45]. The AST (Anxiety, Sweating and Tremor) scale was developed and validated at the Johns Hopkins Bayview Medical Center Chemical Dependency Unit (CDU) in Baltimore, Maryland. The score range of items was extended from 0 to 3 in order to reduce the subjectivity during evaluation, and the initial evaluation and nursing comments were taken into account. The AST scale represents an easy-to-applicate tool in the context of saturated hospitalization services [29] which showed satisfactory concordance with GMAWS and CIWA-Ar scales, suggesting its potential usefulness and reliability to identify subjects with AWS; however, the validation study was carried out in a center of local alcohol detoxification with a population showing low severity of AWS [29], so its validation in a general hospital is desirable. An overview comparison between alcohol withdrawal scales [29,37,38,39,40,41] is shown in Table 1.

In Mexico, there is a high prevalence of general hospital admissions due to AWS, either in Emergency or Internal Medicine Departments. Therefore, it is necessary to look for evaluation scales that are quick and easy to implement, and that help to early identify patients with AWS that require in-hospital surveillance. The objective of this study was to validate the AST scale compared to the CIWA-Ar, in addition to evaluating the diagnostic performance and concordance of the AST scale for the Mexican population with AWS.

## 2. Materials and Methods

A cross-sectional and analytical study was carried out. Inclusion criteria were male gender, age range between 18–65 years old, who were attended at the Emergency Department with a diagnosis of AWS based on the CIWA-Ar scale. Exclusion criteria were head trauma, previous diagnosis of psychiatric disease or previous development of acute delirium, as well as intoxication with substances other than alcohol. Three hospitals of Mexico City were included (Hospital General Xoco, Hospital General Ticomán and Hospital General de Tláhuac) from 1 December 2017 to 30 Jun 2018.

The investigation adheres to the guidelines of the Declaration of Helsinki, Nuremberg Code, and was approved by the institutional ethics committee of Hospital General Xoco (approval no. 207-010-2918). All patients included in the study, or their representative legal entities, signed the informed consent of participation.

All patients were evaluated at hospital admission with the CIWA-Ar, GMAWS and AST scale, with their validated Spanish versions.

### Adaptation of the AST Scale for the Study Population

The English version of the AST scale was translated into Mexican Spanish by bilingual researchers. Subsequently, two physicians who care for these patients adjusted the conceptual definitions to formulate questions appropriately for the evaluation of this group of patients. Cronbach’s alpha test was used to assess the reliability of the AST scale. In addition, factorial analysis was performed, and sensibility and specificity were obtained with an ROC curve with a cut-off point ≥3. Cohen’s kappa statistical test was used to determine concordance.

The degree of severity of the AWS, evaluated by scales (CIWA-Ar, GMAWS, and AST), were compared.

For statistical analyses, Cronbach’s alpha test and Cohen’s kappa were applied to determine reliability and concordance, respectively. Then, factorial analysis and Kaiser–Mayer–Olkin tests were used for homogeneity and evaluation of individual item contributions. Statistics were performed with SPSS v.20, and the Spanish version was used. Statistical significance was considered if *p*-value < 0.05.3.

## 3. Results

Sixty-eight male inpatients diagnosed with AWS were analyzed. Mean age was 41.2 years old, with an education level of high school, without co-morbidities and with robust alcohol consumption for the last 3 months. At admission, vital signs were mean heart rate of 89.3 ± 16.4, systolic blood pressure of 121.6 ± 14.9 mmHg and diastolic blood pressure of 76.9 ± 11.3 mmHg.

At admission, CIWA-Ar, GMWAS and AST scales were estimated for every patient (Table 2).

Initial analysis of age distribution according to the severity of AWS showed no significant differences for the CIWA-Ar (*p* < 0.336) and GMAWS (*p* < 0.078) scales. However, older age was distributed in severe ASW, as evaluated by AST (*p* < 0.04).

For internal consistency, CIWA-Ar (gold standard) and GMAWS showed a reliability of 0.511 (*p* < 0.0001) and concordance of 0.331 (*p* < 0.0001), while consistency for AST was determined as 0.852 and 0.439 (*p* < 0.0001) for reliability and concordance.

According to factorial analysis, GMAWS showed a homogeneity of 0.747, while the profile of components were hallucinations 2.7 (53.4% variance), tremor 0.8 (17.4% variance), orientation 0.7 (13.6% variance), sweat 0.4 (8.0% variance) and anxiety 0.4 (7.6% variance). The AST score showed homogeneity 0.732, and component analysis of tremor was 2.3 (77.5% variance), sweat 0.4 (12.1% variance) and anxiety 0.3 (10.4% variance).

**Figure 1:**
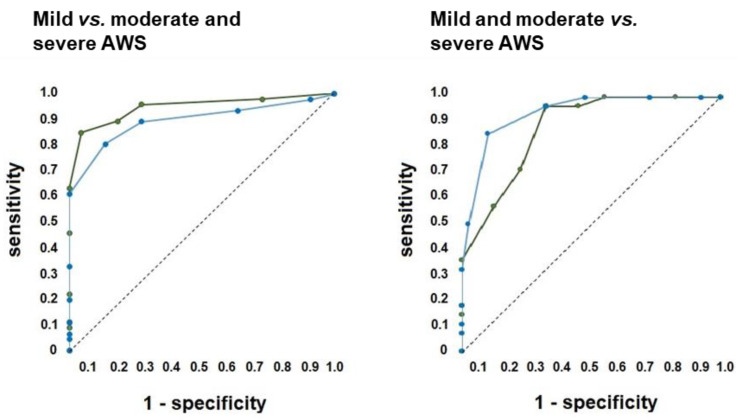
Diagnostic ability of AST scale. The figure shows Receiver Operating Characteristics of AST scale during discriminating mild AWS vs. moderate and severe AWS (**left panel**), as well as mild and moderate AWS vs. severe AWS (**right panel**). AST is shown in blue and GMAWS in green. Abbreviations: AWS, Alcohol Withdrawal Syndrome.

Discriminant performance of AST and GMAWS for different severities of AWS, as reflected by CIWA-Ar, were determined, and results are shown in Figure 1 and Table 3. AST evidenced a similar diagnostic performance as GMAWS.

## 4. Discussion

Lack of early detection of AWS limits an opportune therapy and may lead to a worsening of the clinical status. Therefore, the present study evaluated the comparative potential of the AST scale to reliably identify individuals with AWS in the Mexican population attending public general hospitals.

The main finding was that the AST scale showed an acceptable internal consistency, reliability and concordance, as compared with the CIWA-Ar and GMAWS scales. Diagnostic performance of the AST scale was similar to the GMAWS scale, the former being able to identify subjects who required symptomatic treatment with a sensitivity of 84% and a specificity of 83.3%, as well as a discriminating potential for a CIWA-Ar > 8 cut-off rendering an AUC of 0.837, and a diagnostic profile comparable to the sensitivity of 93% and specificity of 63% initially reported by Holzman and Rastegar [29]. Furthermore, AST’s positive predictive value of 93.3% indicates a high probability to identify severe cases of AWS.

This profile suggests that the AST scale, an easier to applicate tool, may also favor the early diagnosis and care of patients with severe AWS. In addition, during subsequent evaluation of AWS to adjust therapy, the AST scale is more versatile to apply than CIWA-Ar and GMAWS, which require a greater investment of time, representing a potential limitation [29]. Therefore, the findings of the present study suggest that the AST scale is a promising tool for the rapid detection and stratification of AWS in large groups and in the hospital context, which can contribute to timely and adequate patient care, according to the severity of the problem, and for the prevention of its progression to severe symptoms.

In general, the three scales, CIWA-Ar, GMAWS and AST, identified that the older the patient is, the worse the estimation of AWS severity. This finding may be different from the data reported by Holzman and Rastegar [29], probably explained by the mild severity of AWS in the patients from the Baltimore Center. Likewise, AST and GMAWS were able to discriminate cases without AWS from mild AWS, whereas CIWA-Ar may not. One possible explanation is the different weight assigned to items in each scale, as well as the stratification cut-off values, rendering different diagnostic performances.

Some limitations of the present study should be considered, including the low number of participants and the type of population, composed exclusively by males. Such conditions do not allow stratified analyses according to age and gender, which would have been desirable to increase the validity of our results. Future studies with a larger sample size would be convenient, as well as the inclusion of women to increase the diagnostic confidence and statistical power, in such a way that AST utility can be further supported.

AUD and AWS are important health problems in Mexico and worldwide that can give rise to several comorbidities and worsening life quality. It is imperative to develop evaluation tools that are quick and easy to apply to optimize early diagnosis and more opportune therapy. The results of our study contribute to the efforts to validate scales for the evaluation of AWS, particularly in the general population attending public hospitals, which will help to identify its usefulness, efficacy and diagnostic confidence in clinical practice.

## 5. Conclusions

The performance of the AST scale in evaluating AWS is comparable to the commonly used CIWA-Ar and GMAWS scales, with the advantage that AST represents a versatile and easy-to-apply instrument, which may be useful for an early identification and management of AWS in several clinical settings, and particularly in public hospitals.

## Figures and Tables

**Table 1:** Comparison of Alcohol Withdrawal Scales.

	CIWA-Ar	GMAWS	AST
No. Of items	10	5	3
Names of the items	Nausea and vomiting Tremor Paroxysmal sweats Anxiety Agitation Tactile disturbances Auditory disturbances Visual disturbances Headache, fullness in head Orientation and clouding of sensorium	Tremor Sweating Hallucination Orientation Agitation	Anxiety Sweats Tremor
Range of scores	0–7 (one item is scored 0–4)	0–2	0–3
Maximum score	67	10	9
Cut-offs for treatment	≥8	≥1	≥3

Abbreviations: CIWA-Ar, Revised Clinical Institute Withdrawal Assessment Scale; GMAWS, Glasgow Modified Alcohol Withdrawal Scale; AST, Anxiety Sweats Tremor Scale.

**Table 2:** Comparison of scales for AWS.

	n = 68			
Scale	w/o AWS n (%)	w/AWS n (%)		
		Mild	Moderate	Severe
CIWA-Ar Mean Score 17.4 ± 11.2	0 (%)	25 (36.8) Age 43.3 ± 11.8 y-o	15 (22.1) Age 39 ± 11.3 y-o	28 (41.2) Age 40.6 ± 8.7 y-o
GMAWS Mean Score 3.9 ± 2.3	3 (4.4%) Age 43.1 ± 11.5 y-o	25 (36.8) Age 42.9 ± 11.8 y-o	35 (51.5) Age 41.1 ± 10.1 y-o	5 (7.4) Age 35.4 ± 6.5 y-o
AST Mean Score 3.8 ± 2.6	7 (10.3) Age 52.6 ± 7.0 y-o	22 (32.4) Age 41.3 ± 10.3 y-o	29 (42.6) Age 40.8 ± 10.7 y-o	10 (14.7) * Age 52.6 ± 7.0 y-o

(*) Denotes a significant difference between age and severity of AWS. Abbreviations: AWS, Alcohol Withdrawal Syndrome; CIWA-Ar, Clinical Institute Withdrawal Assessment Alcohol Scale Revised; AST, Anxiety, Sweating and Tremor Scale; GMAWS, Glasgow Modified Alcohol Withdrawal Syndrome.

**Table 3:** The discriminant ability of AST and Glasgow Scores for severity of AWS.

	CIWA-Ar Mild vs. Moderate, Severe			CIWA-Ar Mild, Moderate vs. Severe		
	AUC	CI_95%_	*p*-Value	AUC	CI_95%_	*p*-Value
AST	0.946	0.894 to 0.998	<0.001	0.875	0.796 to 0.954	<0.001
GMAWS	0.895	0.820 to 0.970	<0.001	0.938	0.885 to 0.992	<0.001

Discriminant performance of AST and GMAWS scales according to different severity levels of AWS, according to CIWA-Ar. The AST scale showed a diagnostic performance comparable to GMAWS scale. Abbreviations: AST, Anxiety, Sweating and Tremor Scale; AWS, Alcohol Withdrawal Syndrome; CIWA-Ar, Clinical Institute Withdrawal Assessment Alcohol Scale Revised; GMAWS, Glasgow Modified Alcohol Withdrawal Syndrome.

## Data Availability

Datasets analyzed or generated during this study can be requested from the authors.
